# Bioimpedance basics and phase angle fundamentals

**DOI:** 10.1007/s11154-022-09780-3

**Published:** 2023-02-07

**Authors:** Leigh C. Ward, Steven Brantlov

**Affiliations:** 1 School of Chemistry and Molecular Biosciences, The University of Queensland, St Lucia, Brisbane, 4072 Australia; 2 Department of Procurement and Clinical Engineering, Central Denmark Region, Aarhus, Denmark

**Keywords:** Bioielectrical impedance, Bioimpedance, Phase angle, Theoretical background, Equivalent circuits, Modelling and simulation

## Abstract

Measurement of phase angle using bioimpedance analysis (BIA) has become popular as an index of so-called “cellular health”. What precisely is meant by this term is not always clear but strong relationships have been found between cellular water status (the relative amounts of extra- and intracellular water), cell membrane integrity and cellular mass. Much of the current research is empirical observation and frequently pays little regard to the underlying biophysical models that underpin the BIA technique or attempts to provide mechanistic explanations for the observations. This brief review seeks to provide a basic understanding of the electrical models frequently used to describe the passive electrical properties of tissues with particular focus on phase angle. In addition, it draws attention to some practical concerns in the measurement of phase angle and notes the additional understanding that can be gained when phase angle are obtained with bioimpedance spectroscopy (BIS) rather than single frequency BIA (SFBIA) along with the potential for simulation modelling.

## Introduction - Bioelectrical impedance analysis of body composition

Bioelectrical impedance analysis (BIA) has become established over the past four decades as a popular technique for the assessment of body composition [1]. The method was originally developed to provide quantitative prediction of total body water (TBW) [2, 3] and fat-free mass (FFM) or lean body mass (LBM) based on a two-compartment model of the human body [4]. The underlying principle of BIA is based on Ohm’s law that states that the potential difference or voltage across a conductor is directly related to the opposition (resistance) to current flow according to Eq. (1)

1 $$R= \frac{E}{I}$$

where R = resistance (ohm), E = voltage (volts) and I = current (amps). For a simple electrically homogeneous conductive cylinder, R varies proportionally to cylinder length (L) and inversely to cross-sectional area (A)

2 $$Volume\propto \frac{L}{A}$$

Combining and rearranging Eqs. (1) and (2) and introducing a constant (ρ) for the proportionality in Eq. (2) yields

3 $$Volume= \rho \frac{{L}^{2}}{R}$$

where ρ is the resistivity or specific resistance of the conductive material. Equation (3) is often invoked the basis for the BIA technique for the assessment of body composition; the conductive volume of the body, i.e. body water, can be estimated from measurement of the electrical resistance (R) of the body (typically from wrist to ankle) and the conductive length (L) usually represented as its proportional surrogate, standing height or stature. Typically, Eq. (3) is solved by using an apparent resistivity (ρ) value derived, either directly or indirectly by regression, in separate calibration studies in which volume is measured as TBW using a reference method such as deuterium dilution (D_2_O dilution)or as FFM using dual-energy X-ray absorptiometry [5]. Although, the human body is not a simple homogenous cylinder with marked geometric variation in body shape undermining the founding assumptions of Eq. (3), the BIA technique is generally considered to provide clinically acceptable predictions of body composition [6]. Nevertheless, there has been growing recognition that absolute quantitative measurement of body composition is not always of paramount importance in clinical practice; what is often of needed are simply indices of change in body composition or physiology that relate directly to cellular function and health status; thus change in resistance alone without empirical transformation to a body composition measure such as FFM may by informative [7–9]. Resistance, however, is only one of the passive electrical characteristics of biological tissues.^1^

## Electrical properties of biological tissues

The interaction of an electromagnetic wave or force with matter are described by complex conductivity and permittivity. Conductivity is a measure of the amount of current that will flow across tissues due to an imposed electrical field; permittivity is the amount of charge that will be induced at tissue interfaces (membranes) by the electric field. Resistivity is the inverse of conductivity. These parameters can be considered as scalars that are a function of frequency and it is generally assumed that the properties of interacting tissues are linear, isotropic and time independent. The early studies demonstrated the dependence of tissue impedance on frequency and recognised that biological tissues have the capacity for, at the microscopic level, energy storage and dissipation. Through the pioneering work of researchers including Maxwell [10] and Debye [11] and Fricke [12] in the 1920s and 30s understanding of the electrical characteristics of tissues improved along with the appreciation that biological tissues exhibit characteristics of a dielectric material. An applied electrical current to biological tissue influences those components that carry a net electric charge and/or a dipolar electrical moment. The most important charge carriers are the mobile ions in tissue water while the key dipolar moments (the separation of positive and negative charges) are the charged protein and lipid molecules of cell membranes. The conductive characteristic (or conversely resistivity) of biological tissue is due to movement of charge while the polarization of dipoles results in a momentary delay known as a dielectric relaxation. Thus, simplistically, opposition to current flow through biological tissue is defined by electrical conductivity (i.e. resistivity) and dielectric permittivity both of which are frequency dependent. Consequently, measurement of these electrical parameters can be related to underlying tissue characteristics and properties. For a more detailed and comprehensive discussion, the reader is referred to the texts by Rigaud et al. [13] and Foster and Schwan [14].

## Electrical models of tissues

**Fig. 1:**
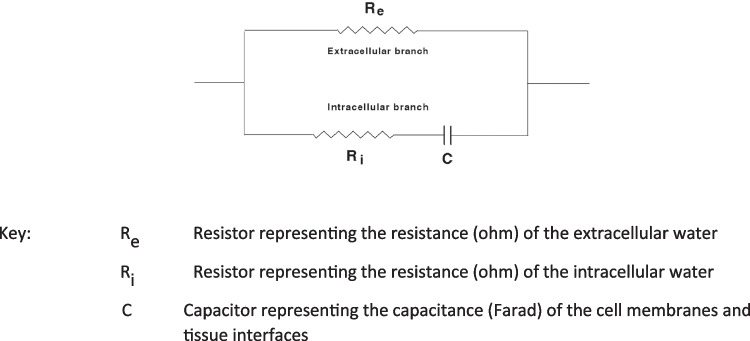
Simple electrical equivalent circuit for biological tissues

When an alternating electrical current passes through the body it will travel through both the extracellular and intracellular fluid compartments in a ratio determined by the frequency of the current and the electrical characteristics of the various tissues. The extracellular pathway is generally considered to be purely resistive whereas the need for the current to pass across the cell membranes which act as imperfect electrical capacitors provide a reactive component. These electrical properties of biological tissues are frequently considered with reference to equivalent electrical circuit models. Many different equivalent circuits have been proposed to describe biological tissues [see 15] although the simplest and most commonly used model is shown in Fig. 1.

This model represents the extracellular pathway of current by a single resistor (R_e_) in parallel with the resistance of intracellular fluid (R_i_) and a capacitor (C) representing the enclosing cell membranes. The impedance (Z) of this equivalent circuit at a specific angular frequency (ω) (where ω = 2πf) is given by

4 $$Z= {R}_{\infty }+ \frac{{R}_{0}- {R}_{\infty }}{1+(j\omega \tau )}$$

where R_0_ is resistance at zero frequency (also named R_e_), R_∞_ is resistance at infinite frequency and tau (τ) is the time constant for a capacitive circuit, i.e., a measure of the rate of accumulation and dissipation of charge. In biological tissues where C is not constant, τ is distributed around a mean value [16] and Eq. (4) becomes

5 $$Z= {R}_{\infty }+ \frac{{R}_{0}- {R}_{\infty }}{1+{(j\omega \tau )}^{(1- \alpha )}}$$

where alpha (α) has a value between 0 and 1 [17]. If the real and imaginary parts of Eqs. (4) or (5) are separated, the resistive (R) and reactive components (Xc) of impedance (a vector quantity) can be defined by

6 $$R= {R}_{\infty }+ \frac{{R}_{0}- {R}_{\infty }}{1+ {\omega }^{2}{\tau }^{2}}$$

7 $$Xc = - \frac{{\omega \tau (R}_{0}- {R}_{\infty })}{1+ {\omega }^{2}{\tau }^{2}}$$

Equations (4) to (7) clearly show that the impedance characteristics of the equivalent circuit and, by extension that of biological tissues, are frequency dependent.

## Frequency dependence of impedance

**Fig. 2:**
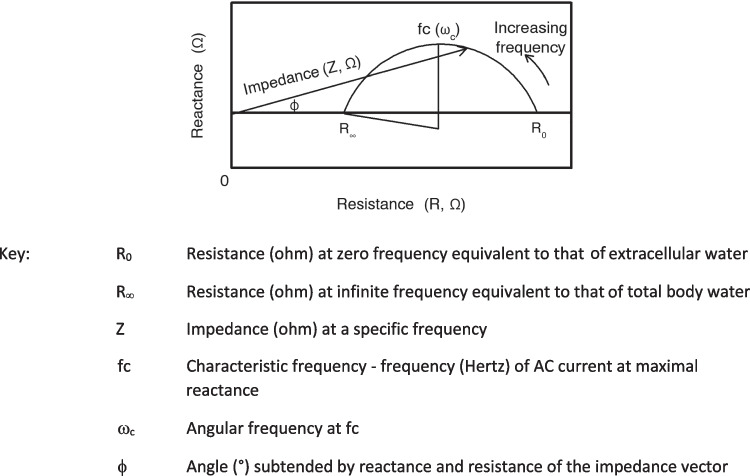
Graphical representation of the frequency dependence of resistance and reactance of a simple circuit analogue of biological tissue with distributed time constants

The relative magnitudes of alternating current flow through the extra- and intracellular branches of the equivalent circuit of Fig. 1 are frequency dependent and are defined by the boundary conditions that at zero frequency the current must pass exclusively through the extracellular resistance (R_e_ = measured resistance or R_0_) since the impedance of the membrane capacitance will be infinite while at infinite frequency the membranes are acting as perfect conductors and the circuit resistance is given by R_∞_ = R_i_R_e_/(R_i_ + R_e_). At any intermediate frequency, current flows proportionately down both parallel branches. This frequency dependence is primarily due to the capacitive nature of cell membranes. A plot of reactance (Xc) against resistance (R) describes a semi-circle (by convention the negative X axis from Eq. (7) is usually ignored and plotted as though positive) or eponymously as a Cole–Cole plot (Fig. 2).

As angular frequency (ω) increases and hence with increasing frequency, the impedance vector traverses anticlockwise; the length of the vector represents the magnitude of the impedance, R and Xc the co-ordinates at the vector tip and phase is represented by the angle (ϕ) between the vector and the resistance (X) axis. For a pure electrical circuit characterized by Eq. (4), the centre of the semi-circular locus lies on the X axis, for biological tissues where there is a distribution of time constants, Eq. (5) pertains and the centre is depressed below the X axis as in Fig. 2.

## Phase angle and frequency dependence of impedance

It is clear from Fig. 2, that, as the impedance vector moves around the semi-circle, phase angle will initial increase with frequency reach a maximum and then decline back to zero. Maximum phase angle is the point of maximum reactance and is defined as the characteristic frequency (ω_c_) where ω_c_ = 2πf_c_. Two important considerations follow from the foregoing analysis. Firstly, that in impedance analysis there is no single phase angle; a phase angle exists at all frequencies of measurement. In common practice, phase angle is *assumed* to be that measured at a frequency of 50 kHz but this is not a formal definition; it is incumbent upon researchers to make clear when referencing phase angle the frequency of measurement. Secondly, the ratio of current flowing down each branch of the parallel circuits in Fig. 1 is independent of capacitance when ω = ω_c_ and is determined by the ratio of R_e_ to R_i_. While this implies that this frequency may be more appropriate for prediction of TBW by Eq. (3) than a fixed frequency of 50 kHz commonly used in single frequency BIA [18], it also suggests that, since reactance is a measure of the dielectric property of cell membranes [4], reactance at ω_c_ is the most appropriate frequency for this purpose. It therefore follows, that if phase angle is to be used as an index of cell mass and membrane integrity it would equally be more appropriately measured at the characteristic frequency.

**Fig. 3:**
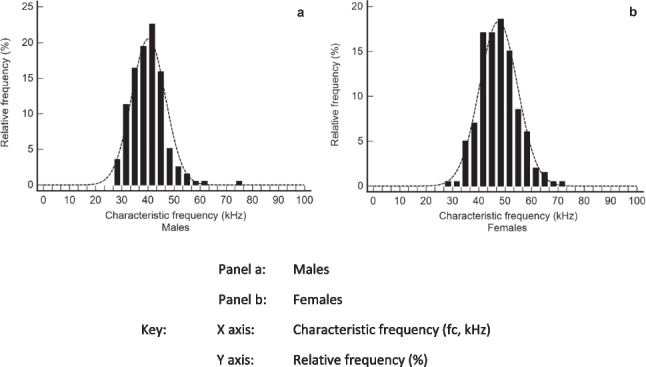
Distribution of characteristics frequencies of a healthy control population. **a** Males, **b** Females Data drawn from a database of impedance data held by the authors. Whole-body impedance measurements obtained in lying with an ImpediMed SFB7 bioimpedance spectroscopy analyser (ImpediMed Ltd., Brisbane). Data analyses and plotted using Medcalc (v 20.115 MedCalc Software bvba, Ostend, Belgium)

In practice however this may not be necessary. Figure 3 shows the frequency distribution of fc values for healthy 394 adults (195 males, 199 females) [19, 20 and unpublished data]. A normal distribution was found with a mean fc of 43.8 kHz (40.2 kHz, males; 47.5 females), very close to 50 kHz the commonly used frequency for phase angle measurements [21]. There was, however, substantial variation in characteristic frequency: an approximately two-fold range for females and an approximately three-fold range for males. The reason for this variation is unclear. Technical errors in determination appear unlikely since at or around maximal Xc, frequency-dependent errors and variability in current flow through intra- and extracellular paths are minimized. Also a similar range in values has been observed by others suggesting it represents primarily biological variation [22]. Since fc is dependent upon the capacitive nature of cell membranes then variations in membrane composition and structure between individuals may be influential. It is also possible variations in tissue anisotropy between individuals are important [23].

**Fig. 4:**
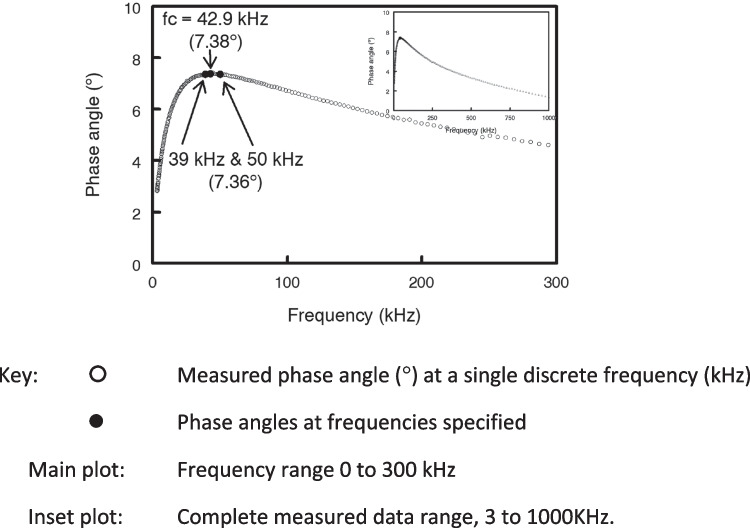
Change in phase angle with frequency of applied current Data for a single male participant drawn from a database of impedance data held by the authors. Whole-body impedance measurements obtained in lying with an ImpediMed SFB7 bioimpedance spectroscopy analyser (ImpediMed Ltd., Brisbane)

Figure 4 shows the change in phase angle with frequency for a typical adult male. Figure 4 inset shows phase angle over the complete measured frequency range with phase angle increasing from zero to a maximum at fc and then declining asymptotically to zero at infinite frequency. Data for measured frequencies from 3 to 300 kHz only are presented in the main plot.

The characteristic frequency for this individual was at 42.9 kHz with a phase angle of 7.38°. Phase angle at 50 kHz was slightly smaller at 7.36°. Consequently little improvement is likely to be seen by using phase angle at fc and avoids the need to use bioimpedance spectroscopy devices in order to determine fc. This observation concurs with only minor improvements in prediction of TBW when using resistance at fc rather than at 50 kHz [24]. It should also be noted that the phase angle observed at 50 kHz is also seen at 39 kHz; only at fc is the value of phase angle unique.

## Measurement of phase angle, some practical concerns

**Fig. 5:**
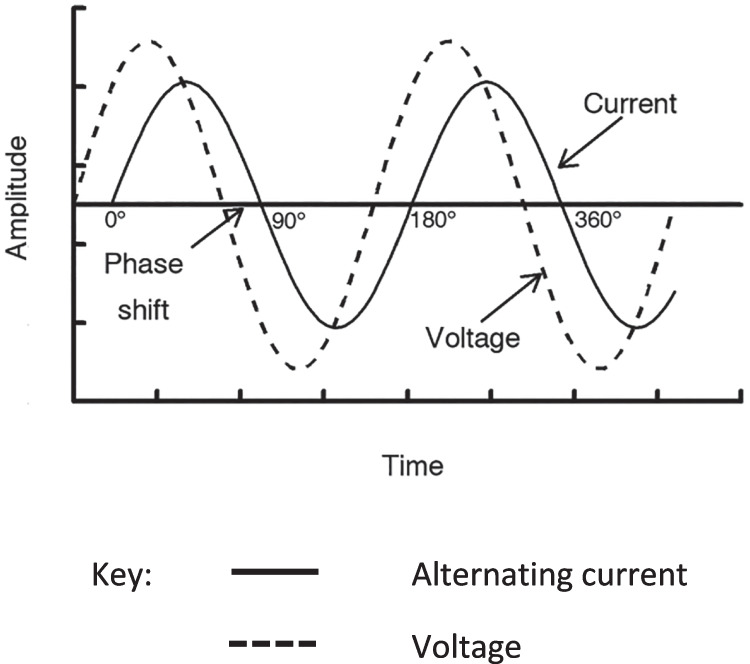
Phase difference in angular waveforms (phasor diagram) due to AC current lagging behind voltage

BIA measurements are obtained using a phase-sensitive electronic instrument. There are a number of different electronic designs that may be used [25] but generally they all have some common features. The device applies a constant low-level alternating electric current to the body via electrodes that span the whole body or a region, e.g., a limb. A pair of proximally-placed electrodes measure the voltage drop as the current flows through the conductive water containing tissues. The attenuation of current flow by the capacitance of the reactive membranes elicits a delay or lag in current flow (Fig. 5).

It is this time delay that is expressed as the phase angle. The precise electronic method by which impedance and phase angle are calculated will depend upon the electronic design of the impedance device and the quality (precision of measurement) of the device componentry. Other than for safety, there are few regulations governing the respective performance of devices from different manufacturers and these may vary with regulatory authority and not all devices may perform similarly [26]. Consequently, the researcher has little control over this other than to use devices that are classed as medical devices or have been independently validated.

In addition, protocols for BIA measurement are not standardized despite its importance being recognized [e.g., 27]. For example, whole-body (hand to foot) impedance measurements may performed with the participant in lying, sitting or supine posture using skin-adhesive Ag-AgCl gel electrodes or for sitting and standing stainless steel contact plates or handles. Different measurement configurations will impact upon the measurements. The separation of the current drive and voltage-sense circuits is designed to mitigate the effects of the electrode-skin interface. However, it requires that electrodes are as identical as practically possible. Nescolarde et al. found large variation in the electrical characteristics of commercially available gel electrodes that significantly impacted measurement of phase angle [28]. This observation has been supported by others in which electrode type significantly impacted both reactance and phase angle [29].

**Fig. 6:**
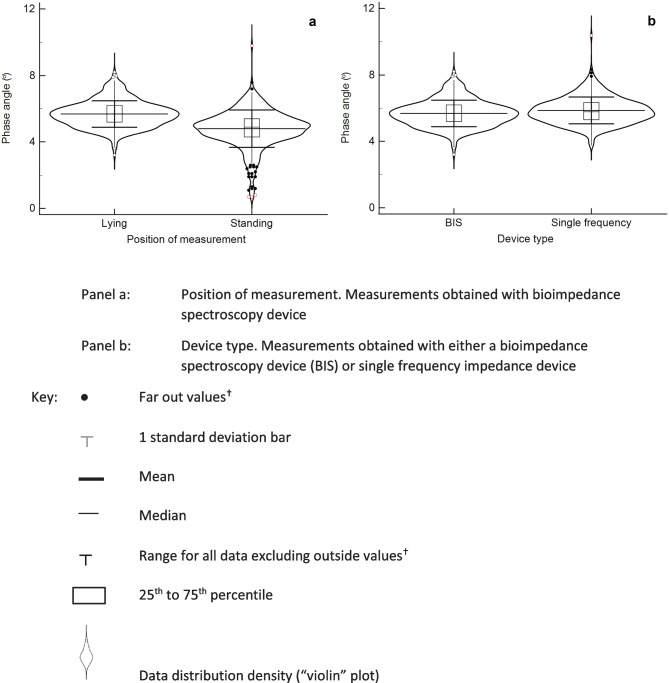
Box and violin plots of phase angles at 50 kHz in healthy males and females combined measured in either lying or standing. **a** Position of measurement. Measurements obtained with bioimpedance spectroscopy device, **b** Device type. Measurements obtained with either a bioimpedance spectroscopy device (BIS) or single frequency impedance device (SFBIA)

The original BIA devices were lead and gel electrode devices and performed measurements with the participant in lying, now stand-on devices are more common where the participant is measured upright. Consequently the effects of gravity and consequent fluid shifts around the body differ between devices. Since phase angle is not only dependent upon the cell membrane capacitance but also the relative proportion of body water in the extra- and intracellular spaces, reflected in R_0_ and R_i_ (Fig. 1) then this is likely to have an impact upon phase angle. The dataset for Fig. 3 provided the opportunity to investigate this. Participants were measured using a lead and electrode device in both lying and standing with the same device. Phase angle was significantly lower (P < 0.001) when measured in standing compared to lying position (Fig. 6a). In addition, data were notably more variable measured in standing. Since the same device was used, only posture changed, these differences are not due to instrumental differences that may confound comparisons between specific lead and stand-on devices. It has also been suggested that phase angle when measured directly at 50 kHz using a single frequency BIA (SFBIA) device may differ from the value determined using a BIS device where phase angle may be determined from data fitting of the complete frequency spectrum [30]. Using the dataset above phase angle was measured in the participants using both an SFBIA device and a BIS device. Both were lead-type devices and the measurements were made in lying. A small (2.9%) but significant difference (P < 0.001) was found (Fig. 6b). This may not however be the case where measured data are very close to the fitted data (data analysis in BIS typically involves fitting the measured data to the semi-circular plot seen in Fig. 2) or where the device provides both sets of values allowing actual measured data to be used. Impedance measurements at 50 kHz have also been reported to be affected by electrical interference from cardiorespiratory monitoring equipment used in a clinical setting [31].

**Fig. 7:**
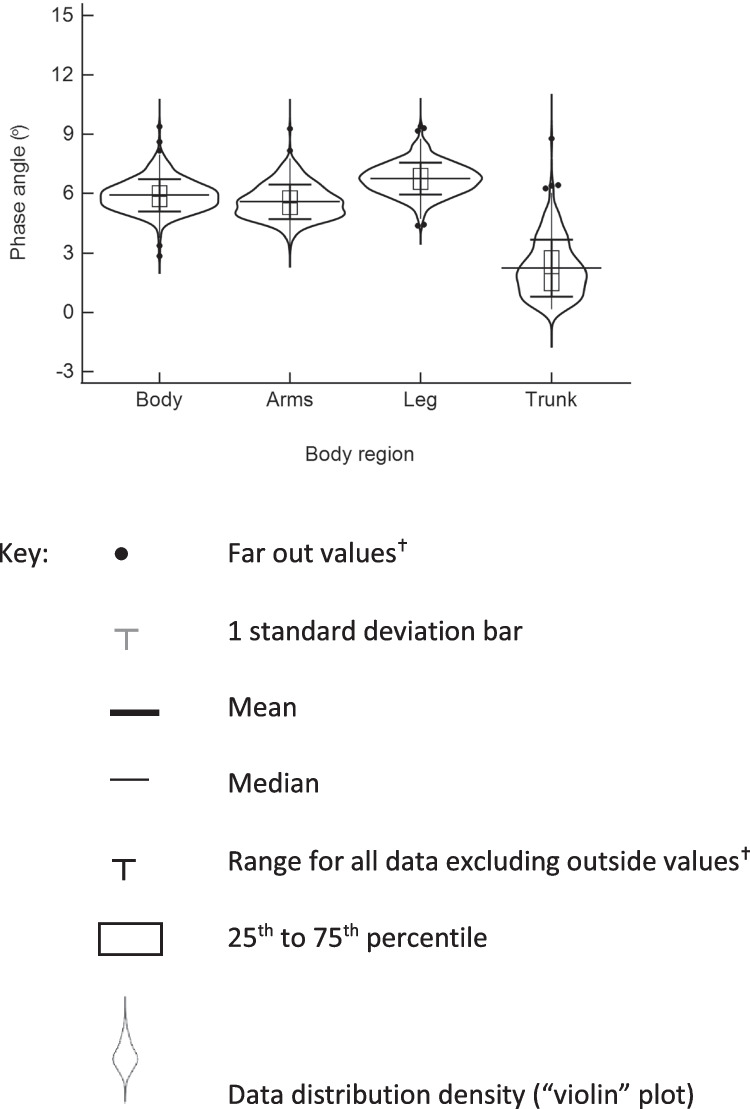
Box and violin plots of phase angles at 50 kHz in healthy males and females combined measured in either lying by body region

The increasingly common stand-on impedance devices are not only more convenient for the participant but also readily provide impedance measurements of the individual body segments not simply the whole body (hand to foot) measurements. This has provided the opportunity to measure phase angle of the separate body regions [32–35] more easily than with SFBIA lead devices (Fig. 7) [36].

These more recent studies have shown that the phase angle of the trunk is consistently lower than that of the other body regions with whole-body phase angle being intermediate between that of the arm and leg unlike early studies that found trunk phase angle was larger than that of the limbs or whole body [36]. This may be a reflection of the technical difficulties associated with truncal impedance measurements. Potentially, segmental phase angles may be more informative of overall cellular health than the body averaged value.

**Fig. 8:**
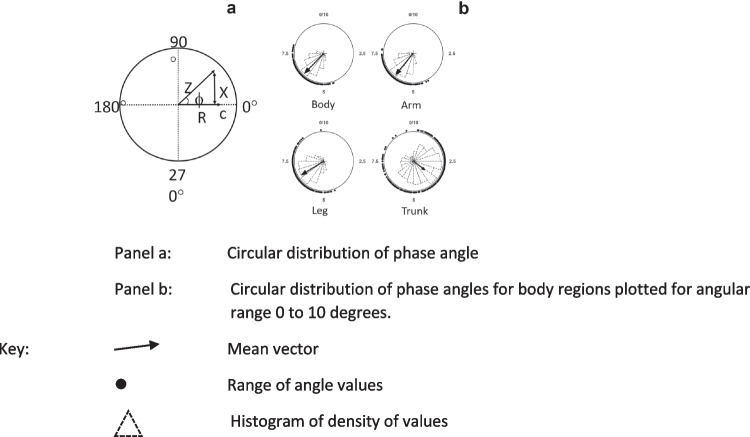
Distribution of characteristics frequencies of a healthy control population. **a** Circular distribution of phase angle, **b** Circular distribution of phase angles for body regions plotted for angular range 0 to 10 degrees Data drawn from a database of impedance data held by the authors. Whole-body impedance measurements obtained in lying with an ImpediMed SFB7 bioimpedance spectroscopy analyser (ImpediMed Ltd., Brisbane). Data analyses and plotted using JASP (v 0.16.4)

By definition, phase angle is an angular measurement with such that the range of phase angles describes a segment of a circular distribution. Since biological phase angles typically fall in a single narrow range they represent a unimodal circular distribution, i.e., a single arc of a circle (Fig. 8a).

It can be argued that such data should be analysed using circular statistical methods rather than the more commonly used frequentist approaches based on a normal distribution. In contrast, circular statistics are based on the von Mises distribution and provides a circular analog of the linear standard deviation [37]. Figure 8b shows the circular distributions for phase angles in the male sample of the data presented in Fig. 7.

## Interpretation of phase angles

**Fig. 9:**
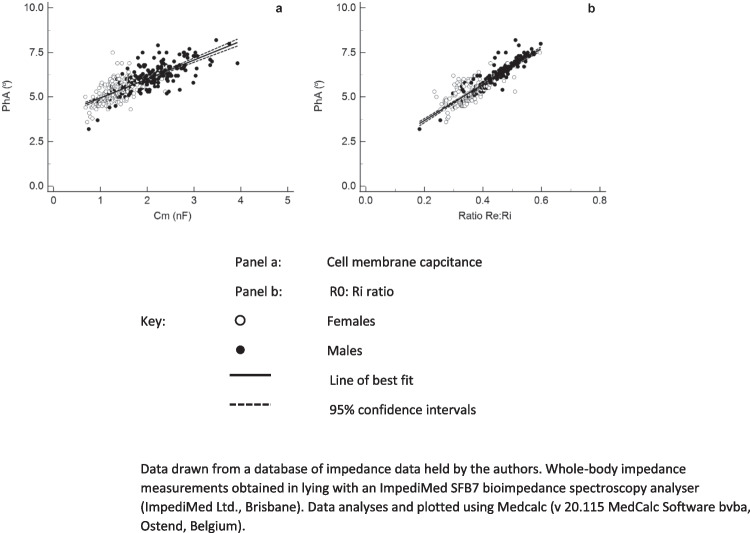
Relationship of phase angle at 50 kHz with cell membrane capacitance and extracellular resistance: intracellular resistance ratio. **a** Cell membrane capacitance, **b** R_0_: R_i_ ratio

It is widely held that phase angle is reflectively of changes in cell membranes and/or the relative amounts of extra- and intracellular fluid. Consideration of the underlying theory outlines above supports this. Studies using BIS also provide empirical support. Figure 9 illustrates the relationship between phase angle and cell membrane capacitance (Cm) and the ratio of extra- to intracellular water represented by the R_e_ (R_0_) and R_i_ ratio. Although variability is present, there are strong correlations for both relationships supporting the view that change in phase angle is acting as a surrogate index of changes in these cellular characteristics. Unfortunately, determining phase angle with a single frequency impedance device only does not allow determination of the relative contributions of either of these cellular parameters to phase angle since neither Cm nor R_e_:R_i_ can be calculated.

The underlying theory of BIA is now well developed and the impedance response of the body can be well modelled by empirical functions such as those of Cole and other researchers [13, 15] although their applicability and accuracy as representative biophysical models has been criticized [38]. Their general acceptance is largely due to their mathematical simplicity and that for all practical purposes they allow interpretation of electrical phenomena in physiological terms although interrelation in terms of biophysical mechanism is considered difficult [6]. Understanding can be advanced by the use of impedance simulation techniques. For example, using the bioimpedance simulator, BioZSim [39], impedance profile of lymphedema, a condition characterized by accumulation of extracellular fluid, is associated with decreases in maximal reactance and increases membrane capacitance [40]. Although not determined in this study, these data suggest that phase angle would consequentially change and was replicable by simulation. Such simulations have the potential to clarify the relationship between cell and tissue structures and impedance measurements, including phase angle. Of particular note is that current models for the analysis of impedance data including phase angle take no account of the anisotropy of biological tissues. In whole body or indeed segmental impedance analysis, the general assumption is that current flow is homogeneous and is predominantly through muscle tissue where current flow is considered to be parallel to the alignment of muscle fibres. This is unlikely, however, to be totally true and investigation of the complex impedance and phase angle of anisotropic tissues may prove fruitful [41].

## Concluding remarks

Measurement of phase angle at 50 kHz has proved to be a useful index of cellular and tissue health in many studies. It is beyond the scope of this introductory review to discuss the potential value and utility of phase angle measurement in health and nutrition research; this is considered elsewhere in this special issue and recent reviews are available [e.g., 35] It is important to recognize that many of these findings are based on empirical observation of associative changes in phase angle with particular clinical or nutritional conditions. Few are theoretically or mechanistically based. As Foster and Lukaski observed in the early days of bioimpedance studies, impedance measurements reflect global characteristics of the body; the connection between impedance and body composition is indirect [6]. These comments were made in the context of using impedance measurements for the quantification of body composition but they are equally applicable to consideration of the biological, physiological or clinical correlates of phase angle. What is now required is to provide a mechanistic explanation for these relationships. This can be facilitated by biomedical engineers, biophysicists and biomedical researchers developing better models that harness present day computational power that was not available to Foster and Lukaski [6].

## Footnotes

1 The BIA method measures the passive electrical properties of cells, tissues or the whole body as a response to an external electrical stimulus. In contrast, some biological tissues, e.g., nerve cells, exhibit active electrical properties in that they are capable of generating voltages and currents. These are *not* measured in BIA.
